# Metabolic response of *Klebsiella oxytoca* to ciprofloxacin exposure: a metabolomics approach

**DOI:** 10.1007/s11306-024-02206-y

**Published:** 2024-12-15

**Authors:** Shwan Ahmed, Sahand Shams, Dakshat Trivedi, Cassio Lima, Rachel McGalliard, Christopher M. Parry, Enitan D. Carrol, Howbeer Muhamadali, Royston Goodacre

**Affiliations:** 1 Centre for Metabolomics Research, Department of Biochemistry, Cell and Systems Biology, Institute of Systems, Molecular and Integrative Biology, University of Liverpool, Liverpool, L69 7ZB United Kingdom; 2 Department of Environment and Quality Control, Kurdistan Institution for Strategic Studies and Scientific Research, Sulaymaniyah, Kurdistan Region, Iraq; 3 Clinical Metabolomics Unit, Institute of Developmental Sciences, University of Southampton, Southampton, UK; 4 Department of Clinical Infection, Microbiology and Immunology, Institute of Infection, Veterinary and Ecological Sciences, University of Liverpool, Liverpool, L69 7BE United Kingdom; 5 Department of Clinical Sciences, Liverpool School of Tropical Medicine, Liverpool, UK

**Keywords:** *Klebsiella oxytoca*, Metabolomics, Sepsis, FT-IR, GC-MS antimicrobial resistance, Metabolic profiling, Metabolic fingerprinting

## Abstract

**Introduction:**

Rapid detection and identification of pathogens and antimicrobial susceptibility is essential for guiding appropriate antimicrobial therapy and reducing morbidity and mortality associated with sepsis.

**Objectives:**

The metabolic response of clinical isolates of *Klebsiella oxytoca* exposed to different concentrations of ciprofloxacin (the second generation of quinolones antibiotics) were studied in order to investigate underlying mechanisms associated with antimicrobial resistance (AMR).

**Methods:**

Metabolomics investigations were performed using Fourier-transform infrared (FT-IR) spectroscopy as a metabolic fingerprinting approach combined with gas chromatography-mass spectrometry (GC-MS) for metabolic profiling.

**Results:**

Our findings demonstrated that metabolic fingerprints provided by FT-IR analysis allowed for the differentiation of susceptible and resistant isolates. GC-MS analysis validated these findings, while also providing a deeper understanding of the metabolic alterations caused by exposure to ciprofloxacin. GC-MS metabolic profiling detected 176 metabolic features in the cellular extracts cultivated on BHI broth, and of these, 137 could be identified to Metabolomics Standards Initiative Level 2. Data analysis showed that 40 metabolites (30 Level 2 and 10 unknown) were differentiated between susceptible and resistant isolates. The identified metabolites belonging to central carbon metabolism; arginine and proline metabolism; alanine, aspartate and glutamate metabolism; and pyruvate metabolism. Univariate receiver operating characteristic (ROC) curve analyses revealed that six of these metabolites (glycerol-3-phosphate, O-phosphoethanolamine, asparagine dehydrate, maleimide, tyrosine, and alanine) have a crucial role in distinguishing susceptible from resistant isolates (AUC > 0.84) and contributing to antimicrobial resistance in *K. oxtytoca*.

**Conclusion:**

Our study provides invaluable new insights into the mechanisms underlying development of antimicrobial resistance in *K. oxytoca* suggests potential therapeutic targets for prevention and identification of AMR in *K. oxytoca* infections.

**Supplementary Information:**

The online version contains supplementary material available at 10.1007/s11306-024-02206-y.

## Introduction

Bacterial infections are a major cause of morbidity and mortality globally (Coates et al., 2002; Kumar et al., 2022a). Early and accurate identification of pathogens and their antibiotic susceptibility is crucial to reduce AMR associated morbidity and mortality caused by bacterial infections. Due to the long time required to obtain antibiotic susceptibility testing (AST) results, broad-spectrum antibiotics are often prescribed as the first-line of medications by clinicians to treat suspected bacterial infections, while awaiting AST results to avoid detrimental outcomes caused by delayed treatment (Patel & Saiman, 2012; Martínez et al., 2020; Li et al., 2023). Unnecessary antimicrobial treatment and misuse of antimicrobial agents are the major causes of the development of antimicrobial resistance by pathogens. The number of resistant pathogens has considerably increased worldwide over the years and it is estimated that AMR will be the cause of 10 million deaths annually by 2050 if actions are not taken (De Kraker et al., 2016). According to the World Health Organization (WHO), the decreasing effectiveness of antibiotics in the 21stst century due to AMR is a serious threat to global health and marks the beginning of a potential post-antibiotic era, where infections associated with minor injuries can lead to death (Organization, 2014; Javad Jafari et al., 2024). The use of antibiotics in veterinary medicine and agriculture has led to environmental contamination and the rise of antibiotic resistance, which has had negative ecological consequences (Polianciuc et al., 2020)

To prevent antibiotic misuse and the emergence of resistant strains during antibiotic treatment, fast and accurate determination of antimicrobial susceptibility of pathogens is needed. However, traditional AST methods used in clinical settings such as disk diffusion, broth microdilution, and agar dilution antibiotics, can take more than 48 h upon sample collection (Benkova et al., 2020). Although real-time polymerase chain reaction (RT-PCR) may be used to infer potential AMR in pathogens by detecting genes associated to resistance, this approach does not provide information about the phenotypic resistance profiles observed in pathogens. By contrast, analytical methods employed in metabolomics approaches represent a good alternative to AST (Kok et al., 2022). These techniques not only enable high-throughput differentiation between resistant and susceptible isolates but also offer a snapshot overview of the underlying biochemical processes associated with resistance mechanisms. MALDI biotyping has emerged as a leading methodology for not only microorganism species identification, but also the detection of antimicrobial resistance in bacterial pathogens within clinical settings. Its rapid and straightforward application make it particularly well-suited for the identification of endemic AMR strains, such as methicillin-resistant *Staphylococcus aureus*, vancomycin-resistant Enterococcus, carbapenem-resistant *Acinetobacter baumannii* and *Pseudomonas aeruginosa*, as well as ESBL-, AmpC-, and carbapenemase-producing Enterobacterales (Yoon & Jeong, 2021). *Klebsiella oxytoca* is a Gram-negative bacterium belonging to the *Enterobacteriaceae* family and is closely related to *Klebsiella pneumoniae*. It is a common cause of nosocomial (hospital-acquired) infections, particularly in critically ill patients, or those undergoing antimicrobial (antibiotic) therapies (Herrera-Van Oostdam et al., 2021; Singh et al., 2016). The *K. oxytoca* complex has the potential to become a major threat to human health due to its ability to cause a wide range of severe diseases, such as septicemia, and its resistance to commonly used antibiotics (Kim et al., 2002; Yang et al., 2022).

Vibrational spectroscopy has been employed as a metabolic fingerprinting tool to detect antimicrobial susceptibility in bacteria in a fast and simple manner, as we have elucidated through our recent research on the antimicrobial resistance profiling of *E. coli* at the individual cell level (Shams et al., 2023) Fourier transform infrared (FT-IR) spectroscopy is a widely used analytical technique that involves the absorption of infrared light by samples under investigation. It is non-destructive, cost-effective, and has high throughput (Confield et al., 2021). The absorption leads to vibrations in the intermolecular bonds, which enable the direct correlation and detection of (bio)chemical species as well as produce unique spectral fingerprints that represent their biochemical composition (Muhamadali et al., 2015). These fingerprints capture specific features related to the structure and composition of biomolecules, including proteins, lipids, carbohydrates, and nucleic acids, within the microorganisms. Javad Jafari et al. used FT-IR spectroscopy to assess the susceptibility of four different bacterial strains to antibiotics by monitoring nutrient consumption in the growth medium as an indicator of metabolic activity (Javad Jafari et al., 2024). A study conducted by da Cunha et al. indicated a strong correlation between the metabolic fingerprints acquired through FT-IR spectroscopy and the mechanisms of action (MOA) of various antibiotics covering a range of targets, including protein synthesis, DNA replication, and cell wall biosynthesis (Ribeiro da Cunha et al., 2020). Furthermore, gas chromatography-mass spectrometry (GC-MS) analysis has also been used for assessing antibiotic susceptibility in bacteria through metabolic profiling. Metabolic profiling aims to detect a wide range of metabolites in a sample and capture all metabolic changes upon exposure to the stressor. It involves the identification and quantification of a specific set of pre-determined metabolites, often associated with a particular metabolic pathway and various analytical techniques can be used for this purpose (Ellis & Goodacre, 2012; Fiehn, 2002). Overton et al. evaluated metabolic profiles of *Salmonella typhimurium* associated with AMR across various hosts through GC-MS analysis (Overton et al., 2022). They demonstrated an increase in the levels of aspartate, alanine, serine and citric acid, while metabolites such as glutamate and pyruvate were shown to bedownregulated upon exposure to different antibiotics. In a different study, Sayqal and colleagues combined both FT-IR and GC-MS analysis to examine the metabolic changes and stress responses of *Pseudomonas putida* strains to toluene. Their findings showed successful discrimination between non-exposed and exposed cultures to toluene and ornithine was identified as the most significant metabolite that increased in response to solvent exposure. Additionally, it was observed that toxic chemical substances were pumped out from bacterial cells into the external environment through the induction of efflux pumps (Sayqal et al., 2016).

This present study aims to assess the phenotype and metabolic response of *K. oxytoca* to antimicrobial challenge using two different techniques, FT-IR spectroscopy and GC-MS, as a metabolic fingerprinting and untargeted metabolic profiling approaches, respectively. The effects of ciprofloxacin on *K. oxytoca* at the metabolomics level were investigated in order to gain a deeper understanding of the metabolic changes observed in susceptible and resistant *K. oxytoca* isolates when exposed to ciprofloxacin. The hypothesis is that non-targeted metabolite profiles will identify biomarker profiles distinctive of AMR between *K. oxytoca* isolates and the identified metabolites will provide sufficient information to determine the key pathways contributing to this discrimination.

## Materials and methods

### Bacterial isolates and growth condition

In this study, six *K. oxytoca* clinical isolates (Table S1) were obtained from children diagnosed with invasive bacterial infection and surveillance within the microbiology laboratory at Alder Hey NHS Foundation Trust, Liverpool, England. *K. oxytoca* isolates were kept as 40% glycerol stocks at -80 °C before being used. Samples were streaked on brain heart infusion (BHI) agar and incubated over night at 37 °C three times to ensure the purity of stocks and availability of axenic colonies for subsequent experiments. Microbial inoculations were prepared from overnight cultures of colonies grown on BHI agar plates.

### Determination of ciprofloxacin minimum inhibitory concentration (MIC)

*K. oxytoca* isolates were inoculated in fresh BHI broth containing different concentrations of ciprofloxacin and their growth profiles were assessed by monitoring the optical density at 600 nm (OD_600_) using a T Bioscreen C spectrophotometer (Oy Growth Curves Ab Ltd., Finland). The full procedure is described in Supplementary Information.

### FT-IR spectroscopy

#### Sample preparation

Following the incubation period, samples were centrifuged at 4 °C for 4 min at 5000 x*g* using a benchtop Eppendorf microcentrifuge 5424R (Eppendorf Ltd., Cambridge, UK). The supernatant was discarded, and the biomass was washed by resuspending it in 1 mL of sterile physiological saline 0.9% NaCl solution, followed by another centrifugation step to eliminate any residues from the media. Finally, the bacterial concentration was adjusted to an OD_600nm_ of 20. There were three biological replicates per condition collected over three days.

A Bruker 96-well silicon sampling plate was cleaned by soaking it in 5% sodium dodecyl sulfate (SDS) overnight, followed by multiple washing steps using 70% ethanol and finally rinsed with deionised water (Muhamadali et al., 2016). Samples were prepared by spotting 20 µL aliquots (randomly located on the plate) of normalised bacterial slurry onto a clean plate followed by incubation to dryness at 42 °C for 45 min in a standard oven.

#### FT-IR instrument setup

The FT-IR bacterial spectra were collected in absorbance mode using a Bruker Invenio infrared spectrometer (equipped with HTS-XT motorised microplate reader) as described in (Winder et al., 2006). The spectra were acquired in the mid-infrared range between 4000–400 cm^-1^ range, with 64 scan co-adds at a 4 cm^-1^ resolution (Muhamadali et al., 2015). Prior to each sample measurement, background spectra of the silicon substrate (position A1) were obtained. A total of 12 FT-IR spectra from each bacterial isolate were collected, comprising three biological and four analytical replicates (four readings from different non-overlapping parts of the same bacterial spot).

#### FT-IR data analysis

All spectra were collected using OPUS software and subjected to pre-processing and multivariate analysis in MATLAB version R2020b (The Mathworks Inc., Natwick, USA). The extended multiplicative signal correction (EMSC) algorithm scaled the spectra, as proposed by Martens et al. (Martens et al., 2003). After scaling, the spectral region associated with CO_2_ vibrations (2400 − 2275, and 680–660 cm^− 1^) was replaced with a linear trend.

Following pre-processing, Principal Component Analysis (PCA) and Principal component-Discriminant function Analysis (PC-DFA) were conducted on the processed spectral data. These statistical techniques are commonly used in FT-IR spectroscopy for data exploration, pattern recognition, and classification. PCA condensed spectral data into uncorrelated principal components (PCs), capturing significant dataset variation. These components were then used as input for the PC-DFA to minimise within-class variance and maximise between-class variance for discrimination among different sample groups. PC-DFA is a supervised method that can also be used as a semi-supervised method. The group structure in this case consisted of biological replicates, rather than differences in the type or level of the labelled substrates utilised for growth. For example, if the grouping structure is based on biological replicates rather than genera or species in the classification of bacteria, it is considered a semi-supervised approach. When PC-DFA is used as a supervised method, a priori knowledge of isolates guides the analysis. A training set (30–50% of spectra per class) is used to build the model, while the remaining data serve as a testing set. Projecting the testing set into the PC-DFA space validates the model’s robustness and checks for overfitting. PC-DFA classifies data using principal components and the experimental class structure. The implementation of this approach involves generating a scatter plot, commonly referred to as a scores plot, as well as a loadings plot. The scores plot of the first two PC-DFA scores allows the visualisation of group differentiation (Gromski et al., 2015), while the loadings plot offers to identify the variables with minimal and maximum impact on the observed variation among groups or classes.

### GC-MS analysis

#### Quenching and extraction of intracellular metabolites

BHI broth (50 mL) was inoculated with a single colony using a 10 µL inoculation loop from strains streaked on BHI agar. There were six biological replicates per condition collected over three days. The samples were then incubated at 37 °C with 200 rpm shaking for 18 h. Next, the cultures grown overnight were mixed with fresh BHI broth and the bacterial density was adjusted to an OD_600_ of 0.1 using a 6705 UV/Vis spectrophotometer (Jenway, UK). The inoculated cultures (with a total volume of culture was 17 mL) was then incubated at 37 °C with shaking at 200 rpm for 1 h. The samples were then exposed to various concentrations of ciprofloxacin (0.3 and 2.0 mg/L) after 1 h into the exponential growth phase. During the mid-exponential phase, which occurred 1 h after antibiotic exposure, 1 mL of culture from each Falcon tube was collected for OD measurement to normalise samples at the final stage of extraction. Another 1 mL of the culture was also taken for the FT-IR analysis. Simultaneously, the rest of the culture solution (15 mL) from each Falcon tube was collected and added to a double volume (30 mL) of 60% cold methanol (-48 ºC previously chilled) and mixed rapidly. This process will quench internal cellular processes without releasing metabolites into the cell medium (Winder et al., 2008). The quenched culture was centrifuged at 4800 x*g* and − 8 ºC for 10 min to collect the cellular mass and the rest was promptly discarded. The pellet was further centrifuged for 5 min to remove any remaining supernatant. The cell pellets and collected supernatants were stored at -80 ºC for metabolite extraction and further analysis.

The extraction process involved using methanol as the solvent and liquid nitrogen. The quenched bacterial pellets were suspended in 500 µL of 80% methanol (-48 ºC) and then transferred to 2 mL tubes. An additional 500 µL of 80% methanol (-48 ºC) was used to wash the original tube to ensure full recovery of the bacterial biomass, and combined with the initial suspension. The resulting mixture was thawed on wet ice, followed by thorough vortexing. After one minute of quick freezing in liquid nitrogen and thawing on wet ice, the samples were vigorously vortexed for about 30 s. To enhance the extraction of metabolites from within the cells, a cycle consisting of freeze-thaw and vortex steps was repeated twice more (three freeze-thaw cycles in total). Then, the suspensions were centrifuged for 5 min at -9 ºC and a speed of 13000 x*g*; subsequently, the supernatants obtained were transferred to clean tubes and kept on wet ice. All extracts were then normalised according to their initial OD_600nm_ using 80% methanol. Pooled quality control (QC) samples were also prepared by combining 120 µL from each sample and mixing thoroughly. The QC mix was then divided into three QC samples, each containing 780 µL of the QC mix (Dunn et al., 2011). For GC-MS analysis, 100 µL of internal standard solution (0.16 mg/mL of succinic-*d*_4_ acid, 0.16 mg/mL lysine *d*_4_ and 0.16 mg/mL glycine-*d*_*5*_ in MS grade water) was spiked to all extracts (samples). All samples were dried for 14 h using a speed vacuum concentrator operated at 35 ºC (Eppendorf 5301 concentrator, Eppendorf, Cambridge, UK).

#### Derivatisation

A two-stage chemical derivatisation process was used, as many classes of metabolites in central metabolism are polar and not volatile. Initially, carbonyl groups were replaced through a methoxyamination reaction followed by a silylation reaction. To eliminate any remaining water condensation, samples stored at -80 ºC were subjected to a speed vacuum concentrator for approximately 90 min. Subsequently, the dried extracts were dissolved in 50 µL of 20 mg/mL *O*-methoxylamine hydrochloride in pyridine. They were then vortexed and incubated at 60 ºC for 30 min using a dry-block heater. Next, 50 µL of *N*-Methyl-*N*-(trimethylsilyl) trifluoroacetamide MSTFA was added to the extracts which were mixed again and incubated at 60 ºC for another half-hour period. Following this step, retention index (RI) solution comprising docosane, nonadecane, decane, dodecane, and pentadecane (0.3 mg/mL of each in pyridine) was introduced before centrifugation at 15,800 x*g* for 15 min. The resulting supernatant (120 µL) was transferred to 2 mL amber glass GC-MS vials fitted with inserts for GC-MS analysis.

#### GC-MS setup and data pre-processing

GC-MS data acquisition and analysis parameters:

GC-MS analysis was conducted using an Agilent 8890 GC instrument equipped with an HP-5MS UI capillary column (30 m x 0.25 mm) in conjunction with an Agilent 7250 GC/Q-TOF and a PAL RTC 120 injector from CTC Analytics. The experimental parameters included a 1 µL injection volume, carrier gas at a flow rate of 1 mL∙min^− 1^ helium with a split ratio of 30:1, an injector temperature set to 280 °C, and an oven temperature program starting at 70 °C (maintained for 4 min), then increasing at the rate of 20 °C∙min^− 1^ until reaching 300 °C (maintained for another 4 min). The overall analysis time of the GC-MS was 19.5 min. The mass spectrum data were collected for the range of 40–600 *m/z* (scan mode) with an acquisition rate of 10 spectra/s and an acquisition time of 100 ms/spectrum. The MS was fitted with an electron ionisation source that was set to 70 eV and a constant emission of 5, along with other specified parameters such as scan speed, source temperature and transfer line temperature. GC-MS chromatograms were acquired using MassHunter software (Agilent). Prior to GC-MS pooled QC samples (*n* = 8) were used to condition the column. These QCs were also run regularly through the analysis as were system blanks which were used for post acquisition quality control purposes.

Following the GC-MS analysis, the data were initially converted to mzXML using Proteowizard software, an MS conversion tool commonly used for converting MS files. Subsequently, these data were subjected to deconvolution processing using MS-DIAL version 4.93, which is a widespread software tool and was launched as a universal program for chromatographic deconvolution of untargeted metabolomics data generated through high-resolution mass spectrometry. The software supports various instruments including GC-MS, as described by (Tsugawa et al., 2015). The deconvolution process involved peak picking, alignment, and area normalisation, which were performed using an internal standard (glycine-*d*_5_) and pooled QC samples as references. Furthermore, prior to performing statistical analysis, quality control samples were utilised to ensure data integrity by assessing and eliminating mass features exhibiting significant deviation within QC samples (Wedge et al., 2011). Typically, in non-targeted metabolomics, the relative standard deviation %RSD is calculated to address variations within QC samples, and values up to 25% RSD are considered acceptable. Values with an RSD greater than 25% were deemed unsuitable as features and consequently eliminated from all samples.

#### Data processing and statistical analysis

MATLAB version R2020b (The Mathworks Inc., Natwick, USA) was utilised for the analysis of all acquired data. Following the deconvolution and QC filtering of the GC-MS, PCA and PC-DFA were conducted on the processed data using MATLAB and compared with FT-IR results. These statistical methods are commonly used for data exploration, pattern recognition, and classification. Simultaneously, the data were submitted to the MetaboAnalyst software to perform data pre-processing (were log_10_ transform and Pareto-scaled), multivariate analysis and pathway analysis. Normalisation was conducted during the deconvolution process in MS-DIAL. The initial dataset comprised 176 features. Following the implementation of a QC filter predicated on the RSD, the number of detected features was reduced to 137. As a result, a total of 137 features were used for further statistical analysis, and among them, 40 metabolites (30 MSI Level 2 and 10 unknown) were observed, which significantly differed between susceptible and resistant bacteria. All metabolites identified as Level 2 annotation were subject to the following criteria in MS-DIAL: The EI similarity and identification score were set to a 75% cut off for any similarity match on the library hits, which only selected metabolites that matched the reference spectra by more than 75%. Additionally, at least two other criteria scores, including retention index similarity, EI similarity, and total scores, were required to exceed 75% for the metabolite to be assigned as Level 2. These rigorous criteria were applied to ensure accurate identification of metabolites, and the combination of these scores was essential to classify an annotation as a true positive, thereby increasing confidence in the identification process (Sumner et al., 2007). The pathway analysis was conducted in MetaboAnalyst, and the library was specifically chosen for the *Klebsiella pneumoniae* subspecies *pneumoniae* MGH 78,578 strain in the Prokaryotes section. The KEGG pathway analysis parameters were configured to include all compounds from the selected pathway library in the reference metabolome section. The pathway enrichment analysis output was visualized as a scatter plot, and the pathway impact from significant features was calculated using a hypergeometric test (Li et al., 2018).

## Results and discussions

### Determination of the minimal inhibitory concentration (MIC) of ciprofloxacin in six isolates of *K. Oxytoca*

All six isolates were challenged with varying concentrations of ciprofloxacin hydrochloride (ranging from 0.001 to 256 mg/L) in order to obtain their MIC based on growth profiles assessed by monitoring OD_600nm_. VS0114 and VS0859 isolates showed no growth inhibition even at the higher concentrations (Figure S1A), therefore being classified as resistant strains. By contrast, the four remaining isolates (VS1520, VS1617, VS1669 and VS2210) exhibited MIC values ranging between 0.1 and 0.5 mg/L (Figure S1B), therefore being classified as susceptible. MIC values obtained for each strain are summarised in Table S1.

### Growth profiles of *K. oxytoca* strains upon exposure to different concentrations of ciprofloxacin at mid-exponential phase

**Fig. 1:**
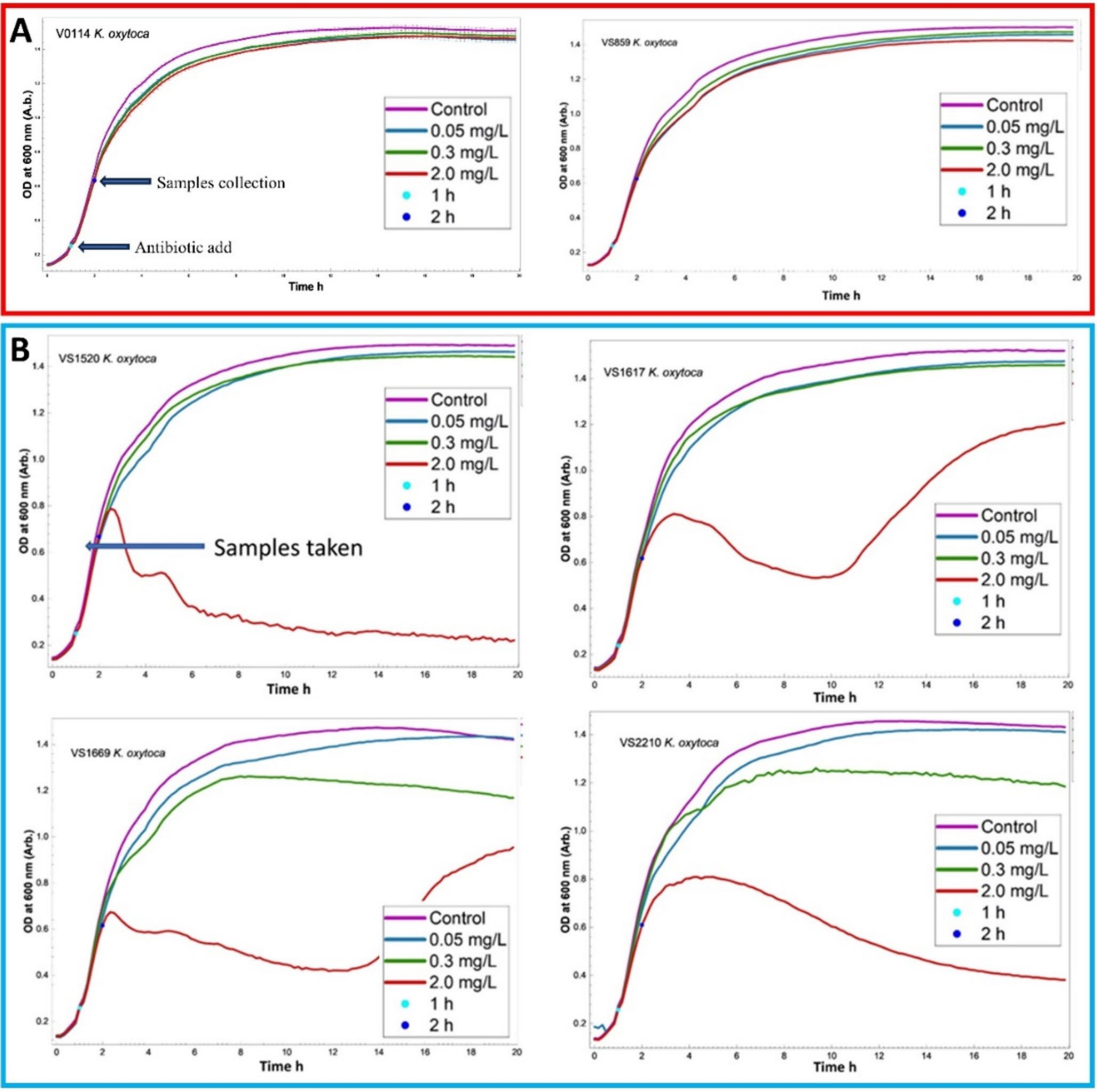
Typical growth curves of the six *K. oxytoca* isolates exhibiting different susceptibilities to ciprofloxacin at three different concentrations. (**A**) Resistant isolates, (**B**) Susceptible isolates. These curves are the means of the five replicates analysed. In (**A**) Error bars represent the standard deviation of five analytical replicates of the first isolate (VS0114), which was reproducible and representative for the other isolates

In order to evaluate the impacts of ciprofloxacin on bacteria, samples were challenged with varying concentrations of 0.01, 0.3, and 2.0 mg/L to represent levels below, within, and above the MIC range, respectively. The growth profiles of all six *K. oxytoca* isolates in this study were monitored in BHI broth medium. According to Fig. 1, no adverse effects on growth patterns were observed within the first two hours of growth (one hour after exposure) for all strains exposed to all three concentrations. This was as expected as the cells were challenged with ciprofloxacin after 1 h of growth in BHI broth, allowing the cell to continue their growth into the start of the exponential phase. At our designated sampling time of 120 min, there was no noteworthy distinction in the growth rate between the control group and the group exposed to ciprofloxacin, and all susceptible and resistant isolates exhibited comparable growth rates and final biomass yields when exposed to ciprofloxacin within the first 2 h of incubation, making it visually difficult to distinguish between them.

The experiment was intentionally designed this way to allow sufficient biomass formation before exposure to antibiotics and to minimise cell lysis and metabolite leakage. However, following this initial period, the growth of susceptible strains was effectively inhibited at 2.0 mg/L, allowing for differentiation from resistant isolates.

### FT-IR spectroscopy of collected *k. oxytoca* biomass samples based metabolic fingerprinting

**Fig. 2:**
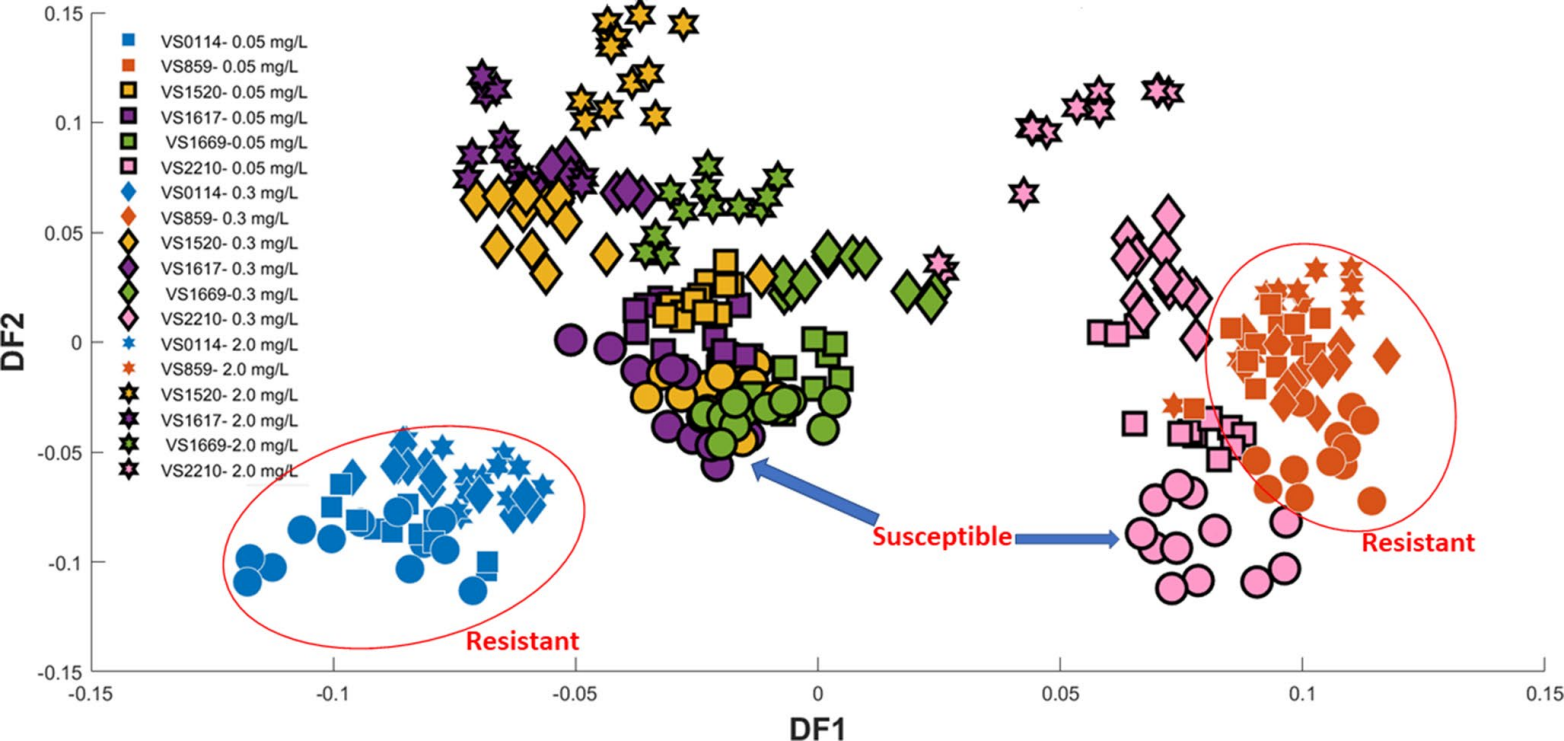
PC-DFA scores plot generated from the first 5 PCs (TEV = 92.78%) showing the relationships of the six isolates of *K. oxytoca* challenged with three different concentration of ciprofloxacin. The untreated group is represented by circles (The black outlined circles represent untreated susceptible isolates, while the non-outlined circles represent untreated resistant isolates). Various symbols refer to different ciprofloxacin concentrations (details provided within the figure)

Here, we evaluated the ability of FT-IR spectroscopy to discriminate susceptible and resistant isolates and assess the effects of different concentrations of ciprofloxacin as an antimicrobial agent. FT-IR spectral data were subjected to PCA, followed by PC-DFA and the clustering patterns were assessed. Figure 2 illustrates PC-DFA scores plot using 5 PCs as input data (total explained variance (TEV) = 92.78%) in a 24 class situation (6 isolates × 4 conditions (untreated and 0.01, 0.3, and 2.0 mg/L-treated)). The metabolic response of susceptible isolates exposed to ciprofloxacin was significantly different compared to resistant isolates as the scores from untreated and ciprofloxacin-treated samples within each resistant strain clustered together. By contrast, susceptible strains displayed a clear trend along DF-2 axis, from untreated to increasing concentrations of ciprofloxacin.

**Fig. 3:**
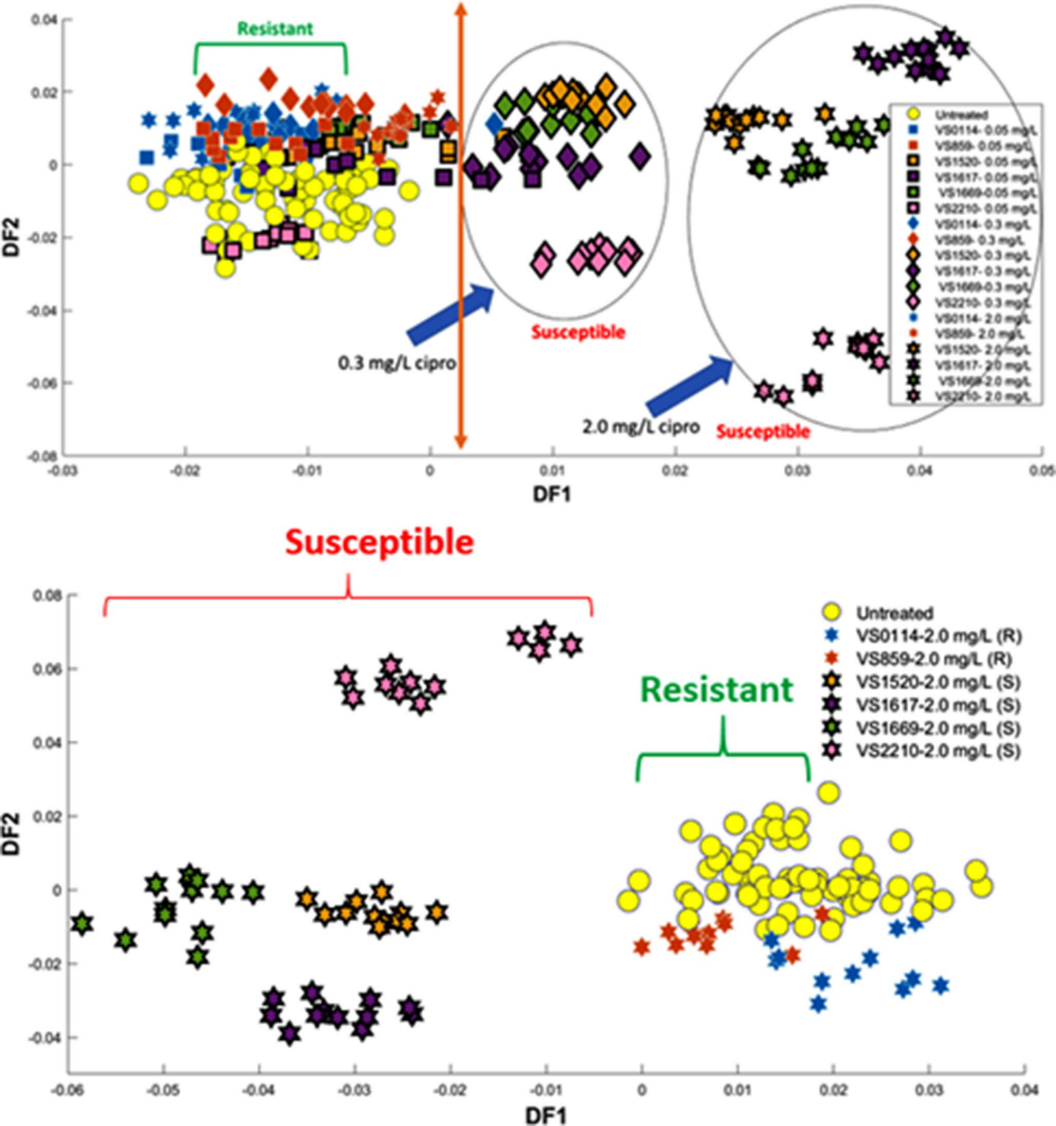
(**A**) PC-DFA scores plot for FT-IR (PCs 1–15, TEV = 98.95%) of the six isolates of *K. oxytoca* challenged to three different concentrations of ciprofloxacin; all untreated isolates were considered as one class (yellow), while various symbols refer to different ciprofloxacin concentrations. (**B**) PC-DFA scores plot for FT-IR data (PCs 1–10, TEV = 97.63%) of six isolates of *K. oxytoca* challenged to 2.0 mg/L of ciprofloxacin. All untreated isolates were considered as one class (yellow)

In addition, we also assessed the clustering patterns observed in the PC-DFA scores plot by considering all untreated samples as one single class. Figure 3A illustrates PC-DFA scores plot of this approach using 15 PCs as input data (TEV = 98.95%). In this plot, untreated samples (control) from all isolates as well as scores from isolates treated with 0.05 mg/L of ciprofloxacin clustered along the negative side of DF-1 axis. By contrast, scores associated with susceptible isolates clustered along DF-1 with a clear trend towards along this axis with increasing concentrations of ciprofloxacin. Interestingly, scores associated to susceptible isolate VS2210 clustered on the negative side of DF-2 axis, while the remaining treated-strains clustered on the positive DF-2, which is associated with the higher susceptibility of this strain to ciprofloxacin as indicated by MIC results (Table S1).

Figure 3B displays PC-DFA scores plot of untreated and samples treated with 2.0 mg/L of ciprofloxacin. Untreated and the treated resistant strains clustered on the positive side of DF-1 axis, while ciprofloxacin-treated susceptible strains were clustered separately on the negative side DF-1axis. In order to reveal the vibrational modes associated with this pattern, we investigated the loadings associated with DF-1 (Figure S2), which displayed the most notable vibrations contributing to the observed clustering patterns and distinguishing susceptible isolates from resistant and control samples across the DF1 axis (Fig. 3). These spectral changes involved peaks at 1021, 1097, 1161, 1242, 1401, 1574, 1626, 1702, and 2928 cm^− 1^ (Figure S2). Based on the vibrational bands mentioned above, the important functional groups that contribute to differentiating susceptible isolates from resistant and control samples displayed higher intensities in alcohols, amides, amines, carboxylic acids, esters, and phosphorus compounds. These molecules are constituents of amino acids, proteins, fatty acids, and phospholipids (Valle et al., 2023). However, their specific concentrations and the sensitivity of FT-IR spectroscopy, in comparison to GC and LC mass-based techniques, limit the ability of FT-IR to provide definitive identification of metabolites in primary metabolism. As such, the functional groups highlighted by FT-IR data can serve as a starting point for designing and conducting further experiments to elucidate the underlying metabolic pathways.

These FT-IR spectroscopy results effectively distinguished the susceptible isolates from the resistant and control isolates at two different ciprofloxacin concentrations; however, this method lacks the required (bio)chemical specificity and sensitivity to identify these significant metabolites. Thus, in order to identify the metabolic response of the cells to these conditions GC-MS analysis was also utilised simultaneously to reach and enhance an in-depth understanding of the metabolic changes in these isolates.

### Metabolic profiling by GC-MS

**Fig. 4:**
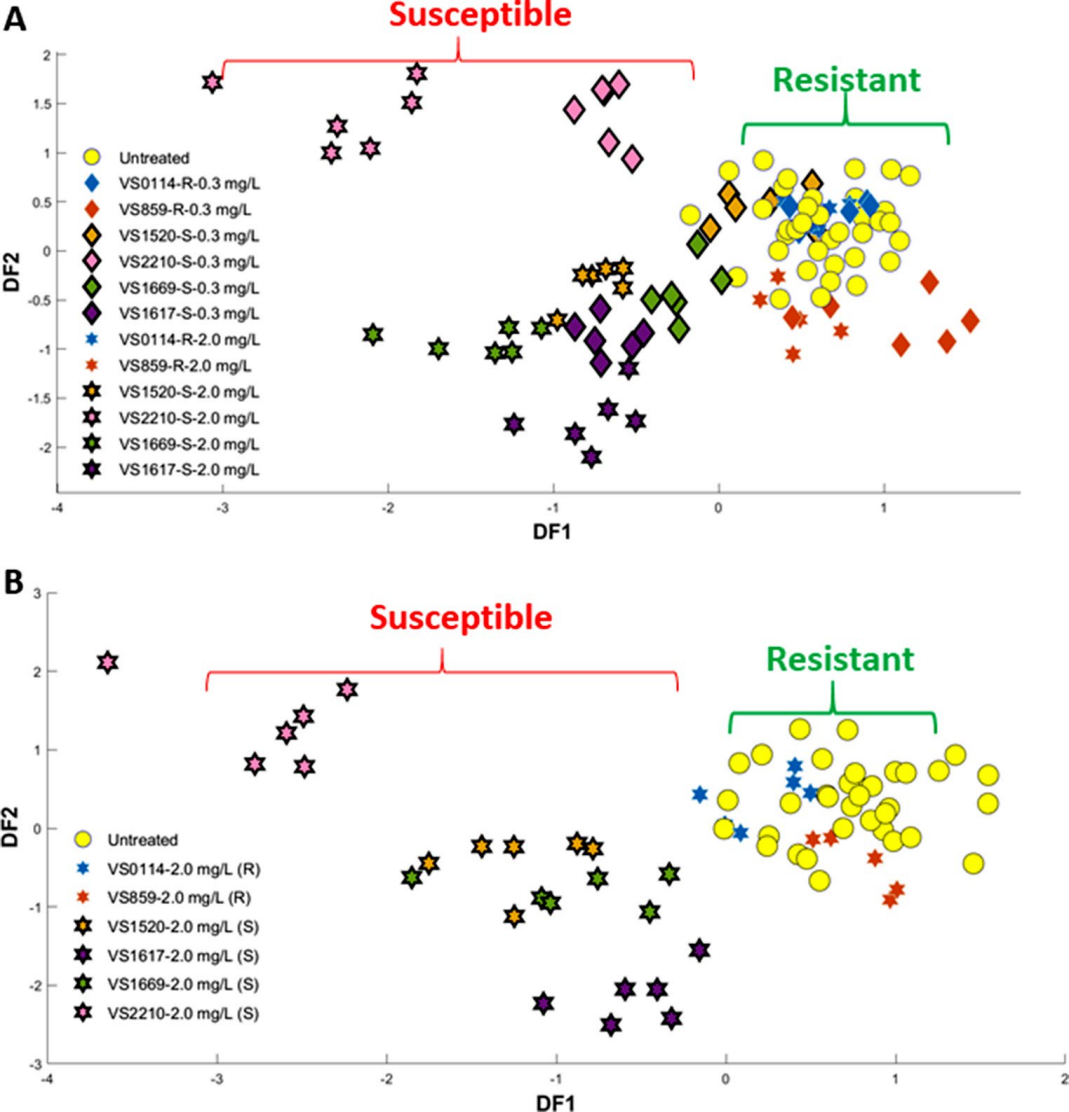
(**A**) PC-DFA scores plot for GC-MS data (PCs 1–30, TEV = 92.45%) showing the relationships between the six isolates of *K. oxytoca* challenged to 0.3 and 2.0 mg/L of ciprofloxacin, All untreated isolates were considered as one class (yellow). (**B**) PC-DFA scores plot for GC-MS data (25PCs, TEV = 91.75%) six isolates of *K. oxytoca* challenged to 2.0 mg/L of ciprofloxacin. All untreated isolates were considered as one class (yellow)

GC-MS analysis was conducted on six clinical *K. oxytoca* strains obtained from sepsis patients to distinguish them based on differences in their metabolic profiles. A total of 137 metabolites were detected following the data pre-processing involving deconvolution, blank filtering, exclusion of internal deuterated standards (lysine, glycine and succinic acid), elimination of false positive peaks, and QC filtering (removing mass features showing significant deviation within QC samples and RSD% values exceeding 25%). Samples were collected for FT-IR and GC-MS simultaneously under similar conditions, allowing us to compare the clustering patterns achieved using both methods. The GC-MS data from all samples were subjected to a multivariate analysis technique (Normalised MS-DIAL data was used for multivariate analysis in MATLAB) to compare it with the clustering patterns observed through FT-IR spectroscopy, and the PC-DFA scores plot was constructed, where 15 principal components (PC), accounting for 92.78% TEV, were utilised as input. The PC-DFA scores plot (Fig. 4A) derived from the cellular extracts demonstrated a clear separation between susceptible and resistant isolates from control samples following exposure to 0.3 and 2.0 mg/L of ciprofloxacin during mid-exponential growth phase. It is clear that the metabolic response to ciprofloxacin differs significantly between susceptible and resistant isolates.

Isolates represented by orange and blue colours and identified as fully resistant based on their MIC values (Figure S1) and growth curves (Fig. 1), were clustered closely with untreated samples at antibiotic concentrations of 0.3 and 2.0 mg/L on the positive side of the DF1 axis. This suggests that there were no significant metabolic changes in the resistant isolates (Fig. 4A). No apparent metabolic distinctions were also observed between antibiotic-resistant and -susceptible strains in the absence of antibiotic exposure. However, a clear trajectory shift from treated to untreated samples (control culture) was observed toward the negative side of the DF1 axis among susceptible isolates with increasing drug concentration. Overall, the findings of the GC-MS analysis aligned with the PC-DFA scores plot of the FT-IR spectral data (Fig. 3A and B).

**Fig. 5:**
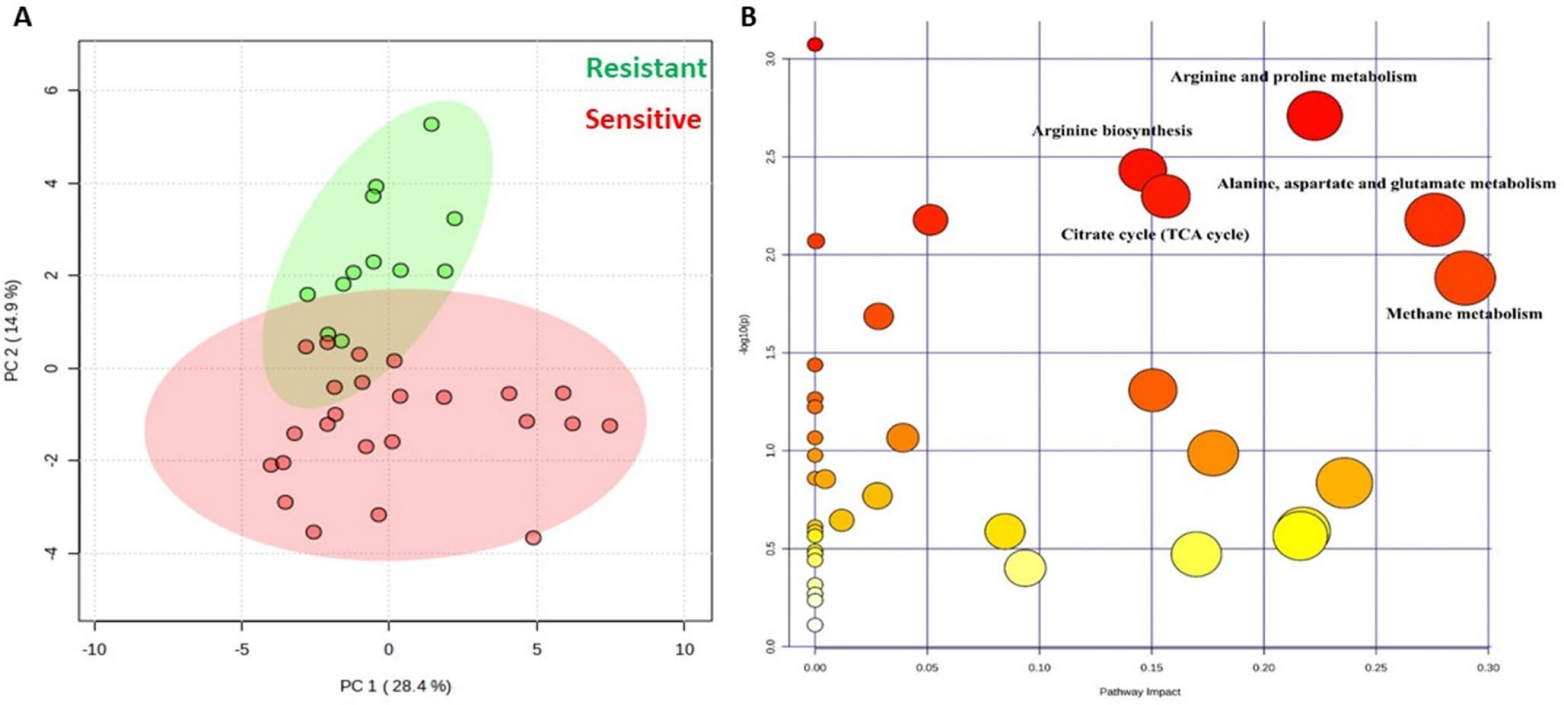
(**A**) PCA scores plot for resistant and susceptible (red) exposed to 2 mg/L of ciprofloxacin and (**B**) The changed metabolic pathways after ciprofloxacin exposure in the six *K. oxytoca* strains are represented by dots, with size indicating pathway impact score and red colour indicating higher significance correlates with the negative logarithm of the *P*-value (-log(*P*) value)

Due to the observed grouping pattern in the PC-DFA scores plot obtained from GC-MS analysis (Fig. 4B) of susceptible and resistant isolates at a ciprofloxacin concentration of 2.0 mg/L, we carried out further investigation using this dosage to understand the potential link between microbial response (difference in metabolite levels) to antibiotics and the observed clustering patterns. Subsequently, the dataset containing 137 detected metabolites was uploaded to the MetaboAnalyst v6.0 platform to perform statistical analysis. Initially, PCA was used to compare the groups and confirm their discrimination (Fig. 5A). A t-test was also conducted to identify significant metabolites contributing to the differences between the two groups based on corrected *P*-value < 0.05 using false discovery rates FDR (statistically significant metabolites). The findings indicated 40 metabolites to be significantly altered in response to ciprofloxacin stress. Among the detected metabolites, 30 were identified at MSI level 2 (putatively identified), matched with database alignment of the measured masses (used a combination of in-house and purchased NIST library), and 10 remained unknown (Sumner et al., 2007). Detailed information on the altered metabolites, including their fold change and *P*-value, can be found in supplementary Table S2. In addition, a pathway analysis was subsequently employed to link metabolites to known metabolic pathways that are active in cells (Smart et al., 2010) and assess the significance of these pathways in the detected biological response (Overton et al., 2022). This serves as a critical method for comprehending the overall impact of antibiotics on pathogens and identifying the key pathways through which bacteria may become susceptible or resistant. To perform pathway analysis, MetaboAnalyst was employed using the 30 known differential metabolites as inputs. The pathway analysis results exhibited major alterations in various metabolic pathways under the ciprofloxacin-treated condition. The effect of ciprofloxacin on *K. oxytoca* was dramatic, as six metabolic pathways displayed notable *P*-values and significant pathway impact upon exposure. The 30 identified intracellular compounds cover various metabolic pathways, including arginine and proline metabolism, arginine biosynthesis, alanine, aspartate and glutamate metabolism, citrate cycle (TCA), methane metabolism, glutathione metabolism, and cysteine and methionine metabolism. However, amongst these the most affected metabolic pathways were arginine and proline metabolism, and alanine, aspartate and glutamate metabolism based on their impact and *P*-values (Table S3).

An overview of pathway analysis is shown in Fig. 5B. These findings are consistent with the literature. For instance, the reduction in alanine, aspartate, and glutamate metabolism pathways on the metabolome of *E. coli* has been documented under fluoroquinolone stress. This decrease has been linked to significant alterations in metabolic activity and considerable enrichment (*P* < 0.01). Furthermore, the application of ciprofloxacin was observed to influence the biosynthesis of aminoacyl-transfer RNAs (tRNAs), leading to an increase in the levels of aminoacyl-tRNA ligases. (Li et al., 2018). Dörries et al. demonstrated that intracellular levels of numerous amino acids in *S. aureus* increased 60 min after exposure to ciprofloxacin. Furthermore, under ciprofloxacin stress, both purine and pyrimidine metabolic pathways were among the most significantly impacted pathways (Dörries et al., 2014).

### Interpretation of the GC-MS metabolite profile data

Metabolomic research has shown the potential to address the issue of AMR through monitoring the modulation of metabolites among pathogens (Kumar et al., 2022b). For instance, combining aminoglycosides with certain metabolic stimuli like fructose and mannitol has been demonstrated to enhance the effectiveness of these antibiotics (gentamicin) in treating *Staphylococcus aureus* and *E. coli* biofilms (Allison et al., 2011). Similarly, fumarate has been found to increase the lethality of tobramycin in metabolically inactive and phenotypically tolerant stationary-phase cells, while glyoxylate conjugation was observed to reduce the activity of aminoglycosides against *Pseudomonas aeruginosa* (Meylan et al., 2017). To date, few investigations have been conducted into the metabolome of *K. pneumoniae* using LC-MS, GC-MS and Nuclear Magnetic Resonance (NMR) spectroscopy (Rees et al., 2017; Foschi et al., 2018; Kumar et al., 2022b). However, to the best of our knowledge, no studies has investigated the metabolic changes in *K. oxytoca* metabolome under ciprofloxacin-induced stress.

We thus aimed to assess the metabolites associated with resistance to ciprofloxacin in *K. oxytoca* isolates, while also investigating the response of the susceptible isolates. Ciprofloxacin is a fluoroquinolone that efficiently inhibits DNA replication by targeting topoisomerase enzymes and is widely used to treat many infections. It acts as a broad-spectrum antimicrobial agent, combating both Gram-negative and Gram-positive bacteria (Castro et al., 2013; Oliphant & Green, 2002).

**Fig. 6:**
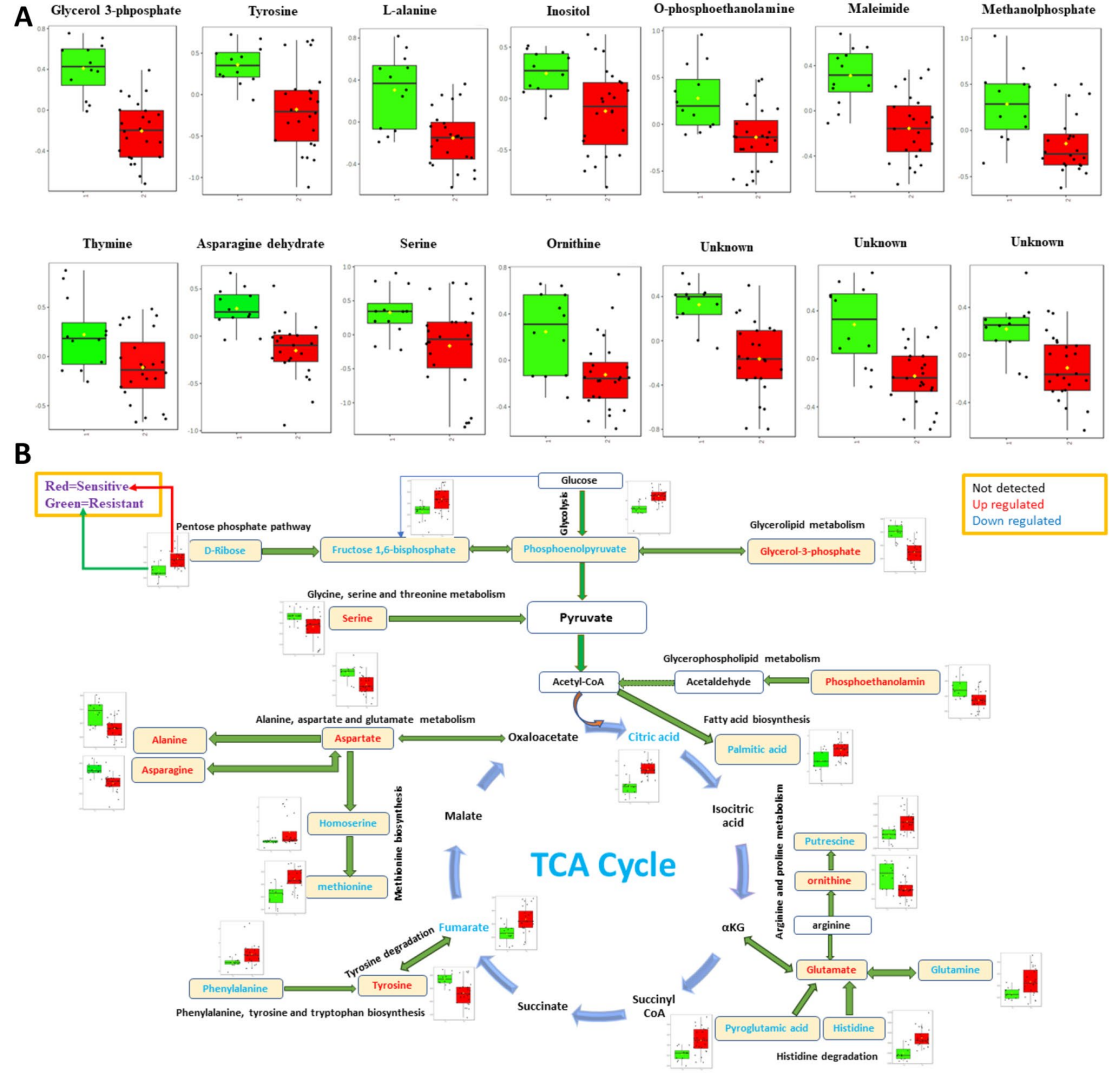
(**A**) Box-and-whisker plots showing up regulated metabolites in resistant isolates challenged with 2.0 mg/L ciprofloxacin concentration and (**B**) Metabolic map of central carbon metabolism and the impact of ciprofloxacin exposure on *K. oxytoca*. Metabolites were identified using GC–MS, with downregulated metabolites shown in blue and upregulated ones in red, while undetected metabolites are represented in black. A box-whisker plot illustrates the alterations in metabolite levels for strains resistant and susceptible (red) to 2.0 mg/L ciprofloxacin challenge. These box and whisker plots depict the interquartile range (edges of the box), the median (middle line in the box) and the black verticle lines represent the whiskers which are the remaining data, with the exception of any data that lie outside the IQR by more than 1.5 × IQR (± 2.7σ). All measurements (including outliers) are represented by black circles

The GC-MS results suggested changes in the relative levels of 40 metabolites were detected which varied between the resistant and susceptible isolates (*P*-value <0.05). Among these, 14 metabolites (including 3 unknowns) were upregulated in resistant isolates (Fig. 6A), while 26 metabolites (including 7 unknowns) were downregulated compared to susceptible isolates (Figure S3).

Glycerol-3-phosphate, maleimide, tyrosine, alanine, inositol, O-phosphoethanolamine, methanolphosphate, serine, ornithine, thymine and asparagine dehydrate displayed higher levels in resistant isolates. In comparison, the level of remaining metabolites decreased in resistant isolates, including those of the TCA cycle and a few amino acids (such as Glutamine, Histidine, methionine and phenylalanine). The GC–MS analysis revealed that more than half the upregulated metabolites in the resistant isolates were also identified in previous study that explored different metabolite extraction protocols and optimised the most effective method for *K. pneumoniae* (Kumar et al., 2022a). These metabolites include Glycerol-3-phosphate, tyrosine, L-alanine, serine, ornithine, and asparagine dehydrate. Additionally, we also detected serine and thymine levels to vary significantly, which were not reported in the previously mentioned studies.

Glycerol-3-phosphate (G3P) is a vital metabolic intermediary in glycerol catabolism, and its expression has notably increased in this study. G3P is transformed into glycerol by phosphatase enzymes present in various organisms (Liu et al., 2022). It plays a key role in the synthesis of triacylglycerides, which are essential lipid components of bacterial cell membranes (Alvarez & Steinbuchel, 2002). Therefore, the level and significance of G3P are closely associated with the rate of bacterial growth and their ability to withstand antibiotic stress.

A key function of enzymes, in the form of amino acids, is to facilitate microbial resistance against antimicrobial agents. Phosphorylation of two amino acids, serine and tyrosine, has been documented to control the binding of proteins that influence DNA repair and replication in *B. subtilis* and *E. coli* (Mijakovic et al., 2006). Our study observed an increase in the relative levels of these two amino acids in the resistant isolates, indicating that the pathogen may elevate their levels to repair the DNA damage and deactivate the mode of action of ciprofloxacin.

Thymine is a crucial component of nucleotides and plays a significant role in the biomolecular functions of living organisms (Bera et al., 2016). It directly affects DNA, causing both single and double-strand breaks. While single-strand breaks can be effectively be repaired, double-strand breaks lead to cell death (Ahmad et al., 1998). Thymine has been proposed as a promising metabolic regulator with the potential to enhance the effectiveness of antibiotics in eradicating Gram-negative bacteria by stimulating bacterial metabolism (Liu et al., 2021). However, the activities of thymine in this study appears to be special. In resistant isolates, the detected thymine level is upregulated, while in susceptible isolates, it is downregulated. This suggests that thymine may not act as a metabolic regulator to enhance antibiotic effectiveness. Instead, the elevated thymine levels in resistant isolates may be attributed to the excretion of excess thymine generated as a result of their survival mechanisms against ciprofloxacin. *O*-phosphoethanolamine (pEtN) is one of the main suppliers of phospholipids, essential components of cell membranes. A reduction in *O*-phosphoethanolamine content can impair the structure and function of bacterial cell membranes. Fluoroquinolones, such as ciprofloxacin, can disrupt bacterial membrane integrity and normal cellular processes due to the induction of oxidative stress. This leads to the generation of reactive oxygen species (ROS) that cause cell damage (V & Veerareddy, 2011). The metabolic response of bacteria, such as *K. oxytoca*, may involve the production of *O*-phosphoethanolamine metabolite as a cellular mechanism to counteract the membrane damage caused by these antibiotics. Our research revealed an increase in *O*-phosphoethanolamine levels in resistant isolates, indicating minimal damage to the cell membrane and potentially contributing to pathogen resistance against antibacterial agents (Zhang et al., 2024). In this study, resistant isolates showed an increase in ornithine levels and a decrease in glutamine levels compared to susceptible isolates. Exposure to ciprofloxacin may enable cells to withstand harsh conditions, leading to an elevation in the production of ornithine as a response to ciprofloxacin stress. Similar to our findings, the upregulation of ornithine and downregulation of glutamine were observed in *P. putida* when exposed to toluene. The decrease in glutamine levels suggests that glutamate could potentially be converted into ornithine instead of glutamine (Sayqal et al., 2016). Maleimide is a cyclic compound containing an imide ring and the CO-N(R)-CO group, which gives it neutral characteristics and hydrophobic properties. It has been demonstrated that maleimide and its derivatives exhibit a range of biological activities, including antibacterial, antifungal, and antitumor properties (Hassan, 2016). However, to the best of our knowledge, the link between maleimide and the effect of ciprofloxacin is not well-established, and there are no reported studies on the role of maleimide in response to ciprofloxacin stress.

The downregulation of putrescine levels in the resistant isolates of *K. oxytoca* were notable, suggesting that these isolates may consume more putrescine as a potential mechanism to reduce the oxidative stress induced by the bactericidal antibiotic such as ciprofloxacin. Putrescine serves as the precursor to polyamines, which are biogenic polycations present in various organisms and are necessary for proper cell division (Igarashi & Kashiwagi, 2000). In *E. coli*, putrescine has been associated with regulating a range of stress response mechanisms, including oxidative stress. The most significant changes in cellular putrescine content (when putrescine was added to *E. coli* cultures) occur in reaction to peroxide-induced oxidative stress and result in enhanced cell survival (Tkachenko et al., 2001). The decrease in citrate and fumarate metabolites levels in energy pathways like the TCA cycle has been observed in antibiotic-resistant strains following antibiotic treatment (Figure S3). This suggests that it could be a potential mechanism for evading antimicrobial or toxicant-induced death by consuming more energy and aiding bacterial survival against antibiotics through the utilisation of efflux pumps, which may require increased TCA cycle activity (Li et al., 2018). Figure 6B presents a comprehensive view of the metabolic map.

Furthermore, in parallel (i.e., without knowledge of the above findings) we conducted receiver operating characteristic (ROC) analysis to characterise the predictive ability of specific metabolites. We identified 15 metabolites with an area under the curve AUC > 0.72. Among them, six upregulated metabolites including glycerol-3-phosphate, *O*-phosphoethanolamine, asparagine dehydrate, maleimide, tyrosine, and L-alanine displayed notably significant AUC values above 0.84 (AUC > 0.84) in resistant isolates. The univariate ROC curve analyses revealed that these six metabolites potentially have a crucial role in distinguishing susceptible from resistant isolates and identifying antimicrobial resistance in *K. oxtytoca* (Figure S4 & S5).

## Conclusions

Metabolic fingerprinting by FT-IR and global metabolic profiling by GC-MS were used to gain insights into the resistance mechanism of *K. oxytoca* when exposed to ciprofloxacin. This work demonstrates how metabolomics is a valuable tool to understand the resistant mechanism and also the response of the susceptible isolates to the drug. The growth curve profiles illustrated that ciprofloxacin had no effect on *K. oxytoca* after exposure to three different concentrations up until the 2 h (the time point for sample collection) from incubation; both susceptible and resistant isolates showed similar growth. The FT-IR data indicates that based on metabolic fingerprints, the PC-DFA scores plots display a clear discrimination between isolates susceptible and resistant to ciprofloxacin at concentrations within and above the MIC level. Furthermore, the PC-DFA plot shows an apparent increase in drug impact with rising ciprofloxacin concentration, leading to enhanced discrimination between them. Analysis of DF1 loadings revealed that various regions containing alcohols, amides, amines, carboxylic acids, esters, and phosphorus compounds contribute to this differentiation. Utilising an FT-IR approach would be beneficial as it allows for rapid analysis of cellular response, enabling more cost-effective and high-throughput experiments. Moreover this method allowed us to establish that there was good reproducibility with respect to the bacterial phenotypic adaptation upon antibiotic exposure as the replicates were congruent. We also conducted GC–MS analysis to observe changes in the metabolome and identify affected pathways. The PC-DFA scores plot showed similar discrimination as seen in FT-IR between the two groups under ciprofloxacin stress. T-test results indicated several significant upregulated metabolites in resistant isolates to combat drug, including glycerol-3-phosphate, tyrosine, *O*-phosphoethanolamine, serine, ornithine and thymine. A decrease in citrate and fumarate levels in the TCA cycle was observed in antibiotic-resistant strains following antibiotic treatment. The most impacted metabolic pathways were alanine, aspartate and glutamate metabolism along with arginine and proline metabolism based on their impact and *P*-values < 0.05. The combination of GC-MS-based metabolic profiling and FT-IR spectroscopic analysis could be a suitable approach for future investigations to better understand the mechanisms of bacterial resistance and identifying antimicrobial resistance in sepsis patients, thereby informing the selection of appropriate antimicrobial therapies.

## Electronic supplementary material

Below is the link to the electronic supplementary material.

Supplementary Material 1

## Data Availability

These data are available via Metabolights with study number MTBLS10502 – https://www.ebi.ac.uk/metabolights/editor/study/MTBLS10502.
