# Correction: Metabolic response of *Klebsiella oxytoca* to ciprofloxacin exposure: a metabolomics approach

**DOI:** 10.1007/s11306-025-02234-2

**Published:** 2025-03-06

**Authors:** Shwan Ahmed, Sahand Shams, Dakshat Trivedi, Cassio Lima, Rachel McGalliard, Christopher M. Parry, Enitan D. Carrol, Howbeer Muhamadali, Royston Goodacre

**Affiliations:** 1 Centre for Metabolomics Research, Department of Biochemistry, Cell and Systems Biology, Institute of Systems, Molecular and Integrative Biology, University of Liverpool, Liverpool, L69 7ZB United Kingdom; 2 Department of Environment and Quality Control, Kurdistan Institution for Strategic Studies and Scientific Research, Sulaymaniyah, Kurdistan Region, Iraq; 3 Clinical Metabolomics Unit, Institute of Developmental Sciences, University of Southampton, Southampton, UK; 4 Department of Clinical Infection, Microbiology and Immunology, Institute of Infection, Veterinary and Ecological Sciences, University of Liverpool, Liverpool, L69 7BE United Kingdom; 5 Department of Clinical Sciences, Liverpool School of Tropical Medicine, Liverpool, UK

**Correction to: Metabolomics**

10.1007/s11306-024-02206-y

In this article, the competing interests section has been updated with the sentence “Prof. Royston Goodacre is the editor-in-chief of Metabolomics”. And it should have been:

**Competing interests:**

The authors declare no competing interests. Prof. Royston Goodacre is the editor-in-chief of Metabolomics.

The original article has been corrected.

