# Supplementary material for: Metabolic response of Klebsiella oxytoca to ciprofloxacin exposure: a metabolomics approach
Source: Metabolomics. 2024 Dec 15;21(1):8. doi: 10.1007/s11306-024-02206-y (PMC11646952; doi:10.1007/s11306-024-02206-y)
Supplement: Supplementary file 1 — Supplementary Material 1 [file 11306_2024_2206_MOESM1_ESM.docx]

**Metabolic Response of Klebsiella oxytoca to Ciprofloxacin Exposure: A Metabolomics Approach**

Shwan Ahmed ^1,2^, Sahand Shams ^1^, Dakshat Trivedi ^1,3^, Cassio Lima ^1^, Rachel McGalliard ^4^, Christopher Parry ^5^, Enitan D Carrol ^2^, Howbeer Muhamadali ^1^, Royston Goodacre ^1^*

1. *Centre for Metabolomics Research, Department of Biochemistry, Cell and Systems Biology, Institute of Systems, Molecular and Integrative Biology, University of Liverpool, Liverpool, L69 7ZB, United Kingdom.*
2. *Department of Environment and Quality Control, Kurdistan Institution for Strategic Studies and Scientific Research, Sulaymaniyah, Kurdistan Region, Iraq*
3. *Clinical Metabolomics Unit, Institute of Developmental Sciences, University of Southampton UK*
4. *Department of Clinical Infection, Microbiology and Immunology, Institute of Infection, Veterinary and Ecological Sciences, University of Liverpool, Liverpool, L69 7BE, United Kingdom.*
5. *Department of Clinical Sciences, Liverpool School of Tropical Medicine, Liverpool, UK*

Corresponding author*: Royston Goodacre

Email: roy.goodacre@liverpool.ac.uk

Keywords: Sepsis, AMR, FT-IR spectroscopy, GC-MS and any other relevant keywords.

**Supplementary Information:**

1. **Brain-Heart Infusion (BHI) broth:**

BHI broth was prepared from a preparatory mixture containing: Brain-Herat Infusion solids (Porcine) 17.5 g/L, tryptose 10.0 g/L, glucose 20.0 g/L, sodium chloride 5.0 g/L and disodium hydrogen phosphate 2.5 g/L. According to the manufacturer's instructions, 37 g of powder was dissolved in 1L of purified water, mixed thoroughly and then autoclaved (at 121 ºC and 15 psi for 15 min).

1. **Comparison of growth curves between susceptible and resistant isolates upon challenged with different concentrations of ciprofloxacin (growth profiles):**

To determine the growth profile of susceptible and resistant isolates, 50 mL of BHI broth was inoculated with single colonies using a 10 μL inoculation loop and incubated at 37 ºC in a shaking incubator at 200 rpm for 18 h. After incubation these overnight cultures were diluted with fresh BHI broth. The bacterial turbidity was adjusted to be equivalent to an OD 0.1 optical density (OD) at 600 nm using a Jenway 6705 UV/Vis. spectrophotometer (Cambridgeshire, UK), prior to further incubation.

A stock solution of ciprofloxacin was prepared and a series of dilutions were made from it (the final concentration of ciprofloxacin in the solutions were 0.05, 0.3 and 2.0 mg/L). 180 μL of these new cultures plus 20 μL of the different concentrations of ciprofloxacin were added to the Bioscreen plate. The negative control samples consisted of 180 µL of new culture and 20 µL of BHI medium. The bacterial growth curves (5 technical replicates) were measured using an OD_600 nm_ in a Bioscreen C spectrophotometer (Thermo Fisher Scientific, Basingstoke, UK). This Bioscreen was run at the following settings: 5 min preheating, measurement interval 10 min, incubation temperature 37 ºC, continuous medium shake and measurements were made for 24 h.

1. **Establishing the minimum inhibitory concentration (MIC):**

MICs were determined using a Bioscreen spectrophotometer through monitoring the growth curves. A stock solution of ciprofloxacin (2 g/L) was prepared in water and a series of dilutions were made from it (final concentration of ciprofloxacin was between 0.001 and 256 mg/L). 10 µL inoculation loops from each isolate (six *K. oxytoca* isolates were cultured on a BHI agar plates) were used to inoculate 50 mL of medium (BHI broth), which was then cultured for 18 hours at 37 ºC in a shaking incubator at 200 rpm. Then overnight cultures were diluted with fresh LB broth. The bacterial turbidity was adjusted to be equivalent to an optical density (OD) of 0.1 at 600 nm using a Jenway 6705 UV/Vis. spectrophotometer (Cambridgeshire, UK). From this new culture, 950 μL with 50 μL of serial dilutions of ciprofloxacin were mixed, and 200 μL of the final solution was added to the Bioscreen plate. The bacterial growth curves were monitored at each condition using a Bioscreen spectrophotometer (Labsystems, Basingstoke, UK). The MIC was determined using five biological replicates, including positive control. The Bioscreen was run at the following settings: 5 min preheating, measurement interval 10 min, incubation temperature 37 ºC, continuous medium shake and mesurements taken for 24 h.

Table S1. Calculated MICs of ciprofloxacin hydrochloride for six isolates of K. oxytoca represent different susceptibilities.

| **Isolate name** | **ID** | \| **MIC range determination (mg/L)** \|  \| \| --- \| --- \| \|  \|  \| | **Source** |
| --- | --- | --- | --- | --- | --- | --- | --- |
| ***K. oxytoca*** | VS0114 | >10.0 | Rectal |
| ***K. oxytoca*** | VS0859 | >10.0 | Blood |
| ***K. oxytoca*** | VS1520 | 0.3-0.5 | Blood |
| ***K. oxytoca*** | VS1617 | 0.3-0.5 | Blood |
| ***K. oxytoca*** | VS1669 | 0.3-0.5 | Blood |
| ***K. oxytoca*** | VS2210 | 0.1-0.2 | Urine |

Table S2 List of 40 metabolites that were differentially expressed between susceptible & resistant samples (p-value <0.05).

| ***m/z*** | **Metabolites** | **t stat** | ***p*-value** | **log10(*p*)** | **FDR*** |
| --- | --- | --- | --- | --- | --- |
| 243.0574 | Glycerol 3-phosphate | 6.401 | 1.21E-06 | 5.9158 | 8.60E-05 |
| 169.0644 | unknown | -5.9406 | 1.26E-06 | 5.9012 | 8.60E-05 |
| 169.0647 | unknown | 5.483 | 4.51E-06 | 5.3458 | 0.000206 |
| 154.0888 | Histidine | -5.3892 | 1.11E-05 | 4.9555 | 0.000379 |
| 174.1092 | Putrescine | -4.7818 | 3.77E-05 | 4.4234 | 0.000837 |
| 154.0287 | Maleimide | 4.9983 | 3.83E-05 | 4.4164 | 0.000837 |
| 273.0915 | Citric acid | -5.3685 | 4.28E-05 | 4.369 | 0.000837 |
| 218.0981 | Tyrosine | 4.483 | 7.96E-05 | 4.0991 | 0.001123 |
| 186.0903 | Heptanoic acid | -4.4948 | 8.19E-05 | 4.0865 | 0.001123 |
| 299.0654 | unknown | -4.668 | 0.000151 | 3.8201 | 0.001495 |
| 210.9959 | Phosphoenolpyruvic acid | -4.7683 | 0.000163 | 3.7872 | 0.001495 |
| 70.06385 | unknown | -4.6498 | 0.000165 | 3.7826 | 0.001495 |
| 176.0887 | Methionine | -4.5337 | 0.000172 | 3.7655 | 0.001495 |
| 156.0808 | Pyroglutamic acid | -4.218 | 0.000175 | 3.758 | 0.001495 |
| 245.0606 | Fumarate | -3.9226 | 0.000482 | 3.3173 | 0.003882 |
| 315.0962 | Fructose 1,6 bisphosphate | -3.7631 | 0.000802 | 3.0961 | 0.005783 |
| 174.1088 | unknown | 3.7947 | 0.000857 | 3.067 | 0.005783 |
| 68.06075 | unknown | 4.0195 | 0.000876 | 3.0577 | 0.005783 |
| 156.0805 | Glutamine | -3.6414 | 0.000914 | 3.039 | 0.005783 |
| 116.0868 | L-alanine | 3.958 | 0.000929 | 3.0322 | 0.005783 |
| 174.1095 | unknown | -3.8701 | 0.001314 | 2.8814 | 0.007573 |
| 252.1304 | 4-hydroxyphenyl acetic acid | -3.58 | 0.001449 | 2.8389 | 0.007941 |
| 217.1025 | Ribose | -3.5459 | 0.001724 | 2.7634 | 0.009086 |
| 140.0862 | Hydroxyproline | -3.4159 | 0.001881 | 2.7257 | 0.009542 |
| 299.0645 | O-Phosphoethanolamine | 3.5075 | 0.002142 | 2.6692 | 0.010479 |
| 103.06 | unknown | -3.2276 | 0.003248 | 2.4883 | 0.014821 |
| 217.0662 | unknown | -3.1691 | 0.003462 | 2.4607 | 0.014821 |
| 241.0426 | Methanolphosphate | 3.3245 | 0.003605 | 2.4431 | 0.014967 |
| 120.0784 | Phenylalanine | -3.053 | 0.00448 | 2.3488 | 0.017682 |
| 243.0922 | Asparagine dehydrate | 3.0943 | 0.004517 | 2.3451 | 0.017682 |
| 204.1192 | Serine | 2.9701 | 0.005472 | 2.2619 | 0.020689 |
| 142.1017 | Ornithine | 3.0876 | 0.005816 | 2.2354 | 0.020968 |
| 179.0596 | Nicotineamide | -2.9503 | 0.006039 | 2.219 | 0.021213 |
| 305.1355 | Inositol | 2.9152 | 0.006648 | 2.1773 | 0.022336 |
| 115.0787 | Creatinine | -2.9369 | 0.006736 | 2.1716 | 0.022336 |
| 218.1341 | Homoserine | -2.9312 | 0.00701 | 2.1543 | 0.022336 |
| 313.2491 | Palmitic acid | -2.9208 | 0.008486 | 2.0713 | 0.026421 |
| 218.0977 | unknown | -2.7757 | 0.012152 | 1.9153 | 0.036997 |
| 248.1261 | unknown | -2.5636 | 0.015184 | 1.8186 | 0.045223 |
| 255.0921 | Thymine | 2.5986 | 0.016523 | 1.7819 | 0.048163 |

*****FDR stands for False Discovery Rate.

Table S3 The results of the pathway analysis highlighting the affected pathways based on their
impact and p-values.

| **Pathways** | **Total** | **Expected** | **Hits** | **Raw *p*** | **log10(*p*)** | **Holm adjust** | **FDR** | **Impact*** |
| --- | --- | --- | --- | --- | --- | --- | --- | --- |
| Arginine and proline metabolism | 31 | 0.57023 | 4 | 0.001953 | 2.7093 | 0.16795 | 0.08495 | 0.22264 |
| Arginine biosynthesis | 18 | 0.3311 | 3 | 0.00369 | 2.4329 | 0.31368 | 0.09626 | 0.14599 |
| Citrate cycle (TCA cycle) | 20 | 0.36789 | 3 | 0.005034 | 2.2981 | 0.42281 | 0.09626 | 0.15626 |
| Glutathione metabolism | 22 | 0.40468 | 3 | 0.006639 | 2.1779 | 0.551 | 0.09626 | 0.05143 |
| Alanine, aspartate and glutamate metabolism | 22 | 0.40468 | 3 | 0.006639 | 2.1779 | 0.551 | 0.09626 | 0.27612 |
| Phenylalanine, tyrosine and tryptophan biosynthesis | 24 | 0.44147 | 3 | 0.008519 | 2.0696 | 0.69 | 0.10587 | 0.00042 |
| Methane metabolism | 28 | 0.51505 | 3 | 0.013143 | 1.8813 | 1 | 0.14294 | 0.28967 |
| Glyoxylate and dicarboxylate metabolism | 33 | 0.60702 | 3 | 0.02062 | 1.6857 | 1 | 0.19932 | 0.02833 |
| Cysteine and methionine metabolism | 46 | 0.84615 | 3 | 0.049131 | 1.3086 | 1 | 0.38858 | 0.15051 |

*The impact on pathways is determined by considering both the centrality of the pathway and the degree of enrichment within the pathway. Higher impact values indicate the relative significance or importance of the given pathway.


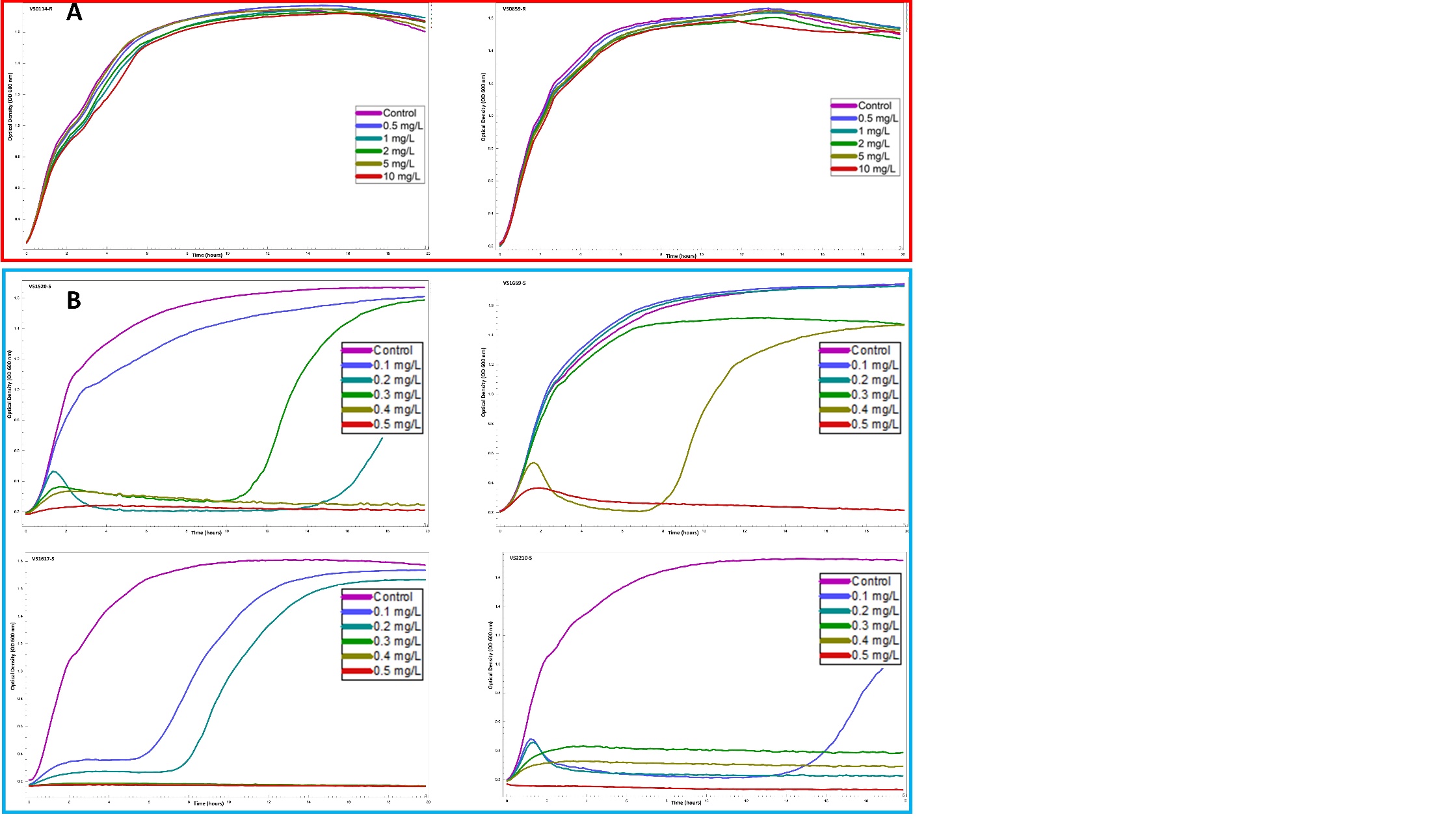


Figure S1. The typicall Minimum Inhibitory Concentration of ciprofloxacin for six K. oxytoca isolates was determined by monitoring the growth curves at OD 600 nm over a period of 0-18 hours after exposure to the antibiotic. (A) two resistant isolates and (B) four susceptible isolates.


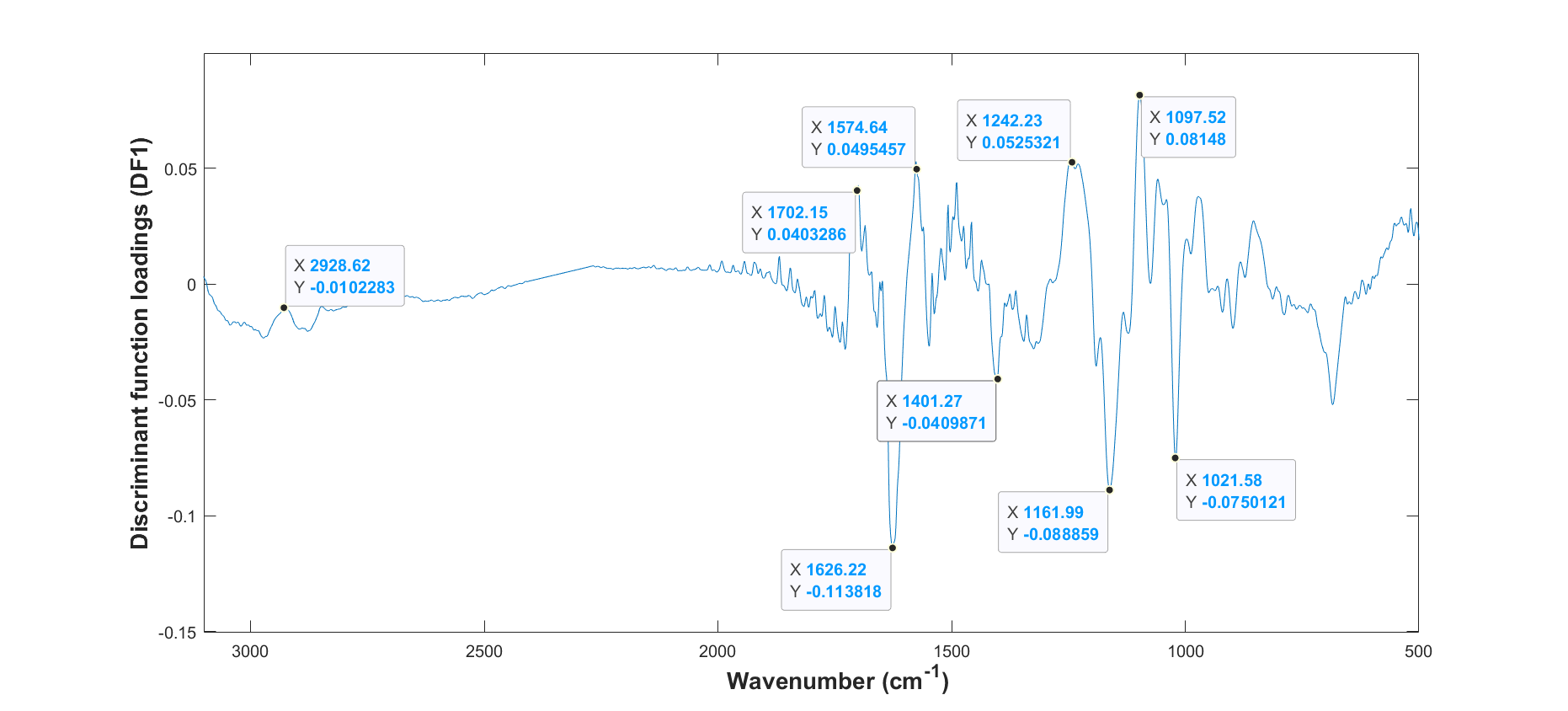


Figure S2. DF1 loadings plot for FT-IR data for six isolates of K. oxytoca challenged to 2.0 mg/L of ciprofloxacin. This corresponds to the PC-DFA scores plots in Figure 3B.


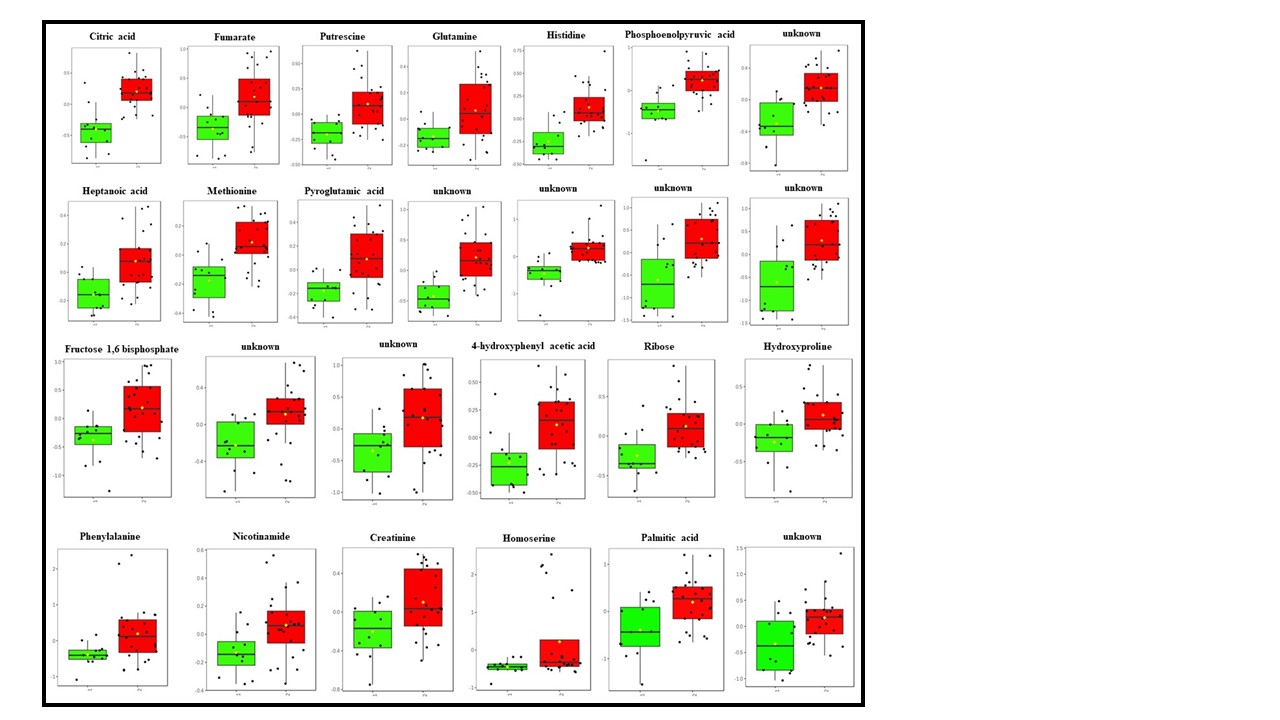


Figure S3. Box-and-whisker plots showing down regulator metabolites in resistant isolates (green) at 2.0 mg/L ciprofloxacin concentration. These box and whisker plots depict the interquartile range (edges of the box), the median (middle line in the box) and the black verticle lines represent the whiskers which are the remaining data, with the exception of any data that lie outside the IQR by more than 1.5 × IQR (±2.7σ). All measurements (including outliers) are represented by black circles.


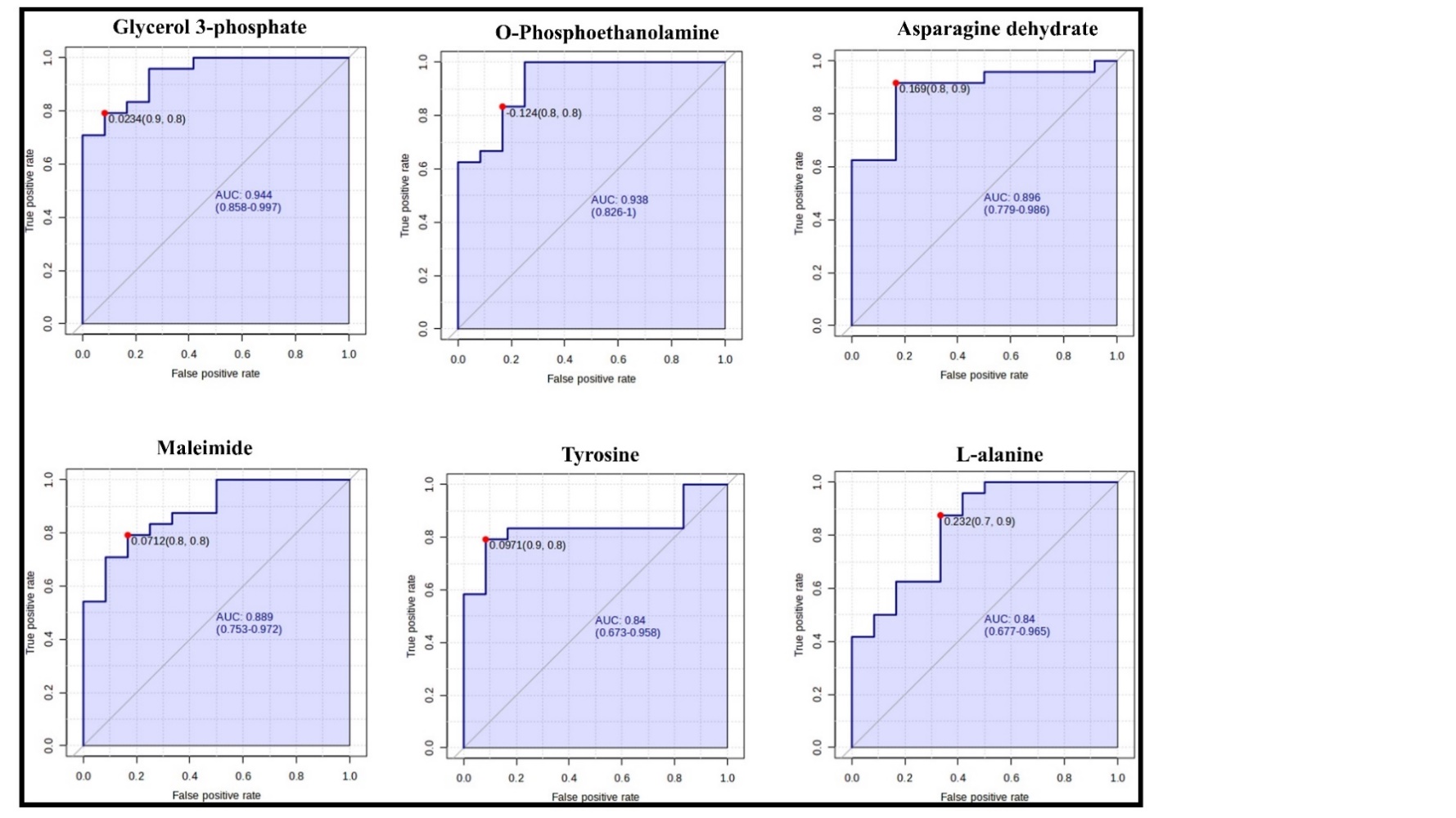


Figure S4. ROC curve analysis of the significant metabolites upregulated for resistant isolates. Glycerol-3-phosphate, O-phosphoethanolamine and asparagine dehydrate demonstrated a high accuracy AUC (> 0.9), high accuracy. Maleimide, tyrosine and L-alanine displayed moderate accuracy with an AUC (>0.84).


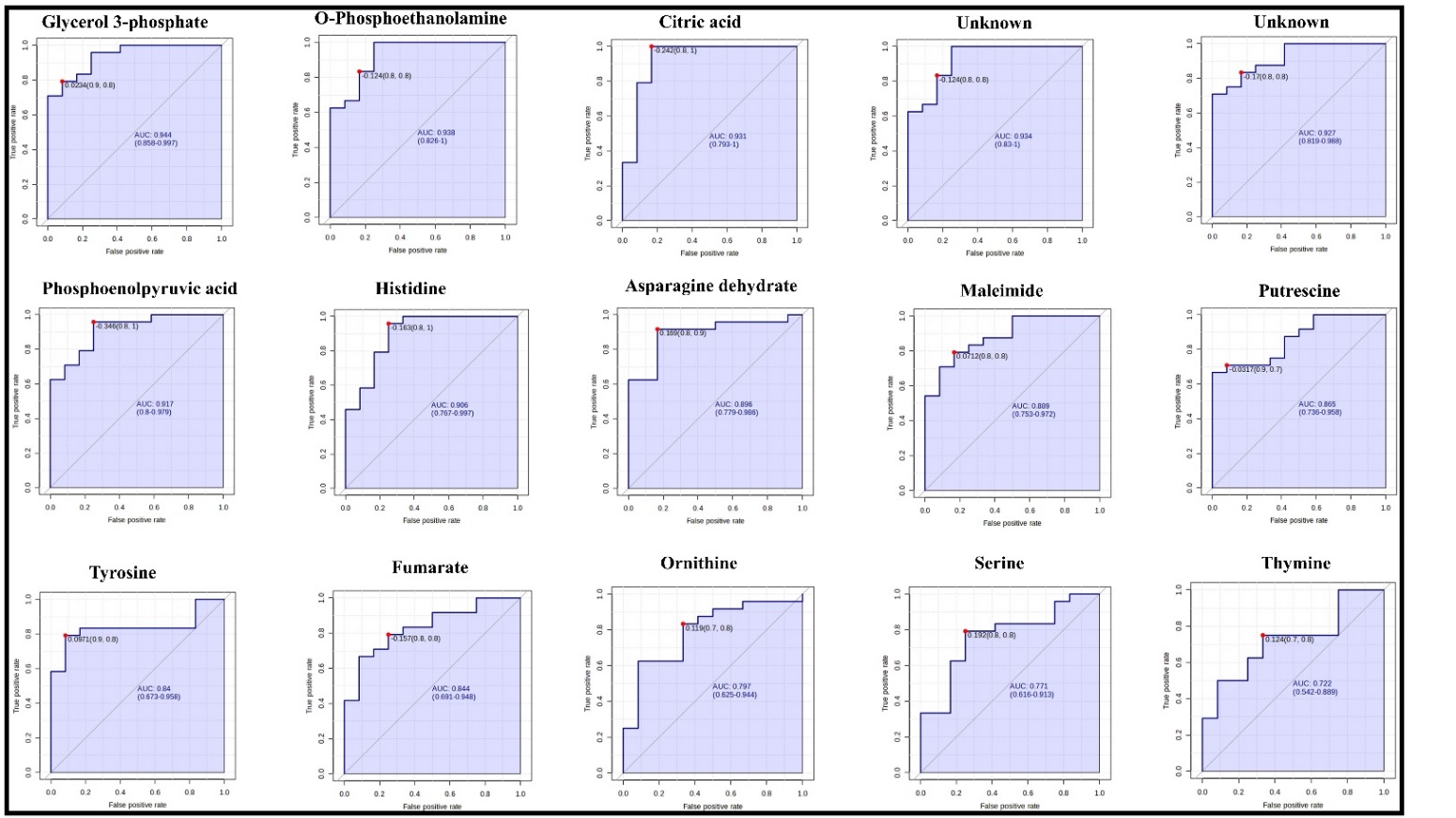


Figure S5. ROC curve analysis of the 15 significant metabolites in discriminating susceptible from resistant isolates with an AUC (> 0.72).
